# 3D printing of anatomically realistic phantoms with detection tasks to assess the diagnostic performance of CT images

**DOI:** 10.1007/s00330-020-06808-7

**Published:** 2020-03-28

**Authors:** Gracia Lana Ardila Pardo, Juliane Conzelmann, Ulrich Genske, Bernd Hamm, Michael Scheel, Paul Jahnke

**Affiliations:** 1 Department of Radiology, Charité – Universitätsmedizin Berlin, Freie Universität Berlin, Humboldt-Universität zu Berlin, and Berlin Institute of Health, Chariteplatz 1, 10117 Berlin, Germany; 2 Department of Neuroradiology, Charité – Universitätsmedizin Berlin, Freie Universität Berlin, Humboldt-Universität zu Berlin, and Berlin Institute of Health, Chariteplatz 1, 10117 Berlin, Germany

**Keywords:** Tomography, X-ray computed, Phantoms, imaging, Health physics, Neck, Radiation protection

## Abstract

**Objectives:**

Detectability experiments performed to assess the diagnostic performance of computed tomography (CT) images should represent the clinical situation realistically. The purpose was to develop anatomically realistic phantoms with low-contrast lesions for detectability experiments.

**Methods:**

Low-contrast lesions were digitally inserted into a neck CT image of a patient. The original and the manipulated CT images were used to create five phantoms: four phantoms with lesions of 10, 20, 30, and 40 HU contrast and one phantom without any lesion. Radiopaque 3D printing with potassium-iodide-doped ink (600 mg/mL) was used. The phantoms were scanned with different CT settings. Lesion contrast was analyzed using HU measurement. A 2-alternative forced choice experiment was performed with seven radiologists to study the impact of lesion contrast on detection accuracy and reader confidence (1 = lowest, 5 = highest).

**Results:**

The phantoms reproduced patient size, shape, and anatomy. Mean ± SD contrast values of the low-contrast lesions were 9.7 ± 1.2, 18.2 ± 2, 30.2 ± 2.7, and 37.7 ± 3.1 HU for the 10, 20, 30, and 40 HU contrast lesions, respectively. Mean ± SD detection accuracy and confidence values were not significantly different for 10 and 20 HU lesion contrast (82.1 ± 6.3% vs. 83.9 ± 9.4%, *p* = 0.863 and 1.7 ± 0.4 vs. 1.8 ± 0.5, *p* = 0.159). They increased to 95 ± 5.7% and 2.6 ± 0.7 for 30 HU lesion contrast and 99.5 ± 0.9% and 3.8 ± 0.7 for 40 HU lesion contrast (*p* < 0.005).

**Conclusions:**

A CT image was manipulated to produce anatomically realistic phantoms for low-contrast detectability experiments. The phantoms and our initial experiments provide a groundwork for the assessment of CT image quality in a clinical context.

**Key Points:**

• *Phantoms generated from manipulated CT images provide patient anatomy and can be used for detection tasks to evaluate the diagnostic performance of CT images.*

• *Radiologists are unconfident and unreliable in detecting hypodense lesions of 20 HU contrast and less in an anatomical neck background.*

• *Detectability experiments with anatomically realistic phantoms can assess CT image quality in a clinical context.*

**Electronic supplementary material:**

The online version of this article (10.1007/s00330-020-06808-7) contains supplementary material, which is available to authorized users.

## Introduction

Adequate image quality is the basis for a reliable diagnosis in computed tomography (CT). This means that the sum of all features of an image must enable radiologists to make an informed judgment. Yet, frequently used image quality metrics such as contrast-to-noise ratios (CNRs) only evaluate selected image features and not how well an image is suited altogether to make a diagnosis. Such metrics can therefore misleadingly indicate high image quality, although diagnostic image performance is actually lower [1]. Image performance has gained in importance with the advent of iterative reconstruction (IR) techniques, which not only reduce noise very effectively but also affect image texture and spatial resolution, so that diagnostic performance can be compromised [2–4]. In order to evaluate the diagnostic quality of an image in terms of how well it enables radiologists to perform diagnostic tasks, appropriate methods should ideally also test how well radiologists can perform diagnostic tasks with the image.

Detectability experiments are such a method. They assess image performance by testing how well detection tasks that are similar to clinical diagnostic tasks can be performed [5, 6]. Most previous studies performed such experiments with low-contrast lesions in uniform phantoms. In other words, they performed detection tasks that mimicked the work of radiologists, but used uniform phantoms that did not. This limitation is of relevance because the texture of phantoms affects the detectability of low-contrast lesions. A previous IR study therefore concluded that image quality should be assessed in the most realistic clinical context possible, i.e., ideally with CT images of patients [7].

3D printing provides novel opportunities to produce phantoms meeting such requirements. Previous work used 3D printing to create low-contrast lesions in cylindrical phantoms [7]. However, no previous work attempted to create low-contrast lesions in phantoms that mimic patient anatomy. The present work therefore used radiopaque 3D printing, a method that was previously shown to provide flexibility and anatomic detail in producing patient-mimicking phantoms [8, 9]. With this method, phantoms representing a patient’s neck and containing different low-contrast lesions were created. The phantoms were evaluated and used in a detectability experiment. The overall aim was to develop anatomically realistic phantoms with low-contrast lesions for detectability experiments.

## Methods

### Study design

The institutional ethics committee approved the study and waived informed consent. Five phantoms with different low-contrast lesions were produced from a CT image of a patient’s neck. The phantoms were scanned with different CT settings and lesion contrasts were measured. Seven radiologists performed a two-alternative forced choice experiment to evaluate how detection accuracy and diagnostic confidence are affected by lesion contrast in anatomically realistic phantoms.

### DICOM data manipulation

**Fig. 1:**
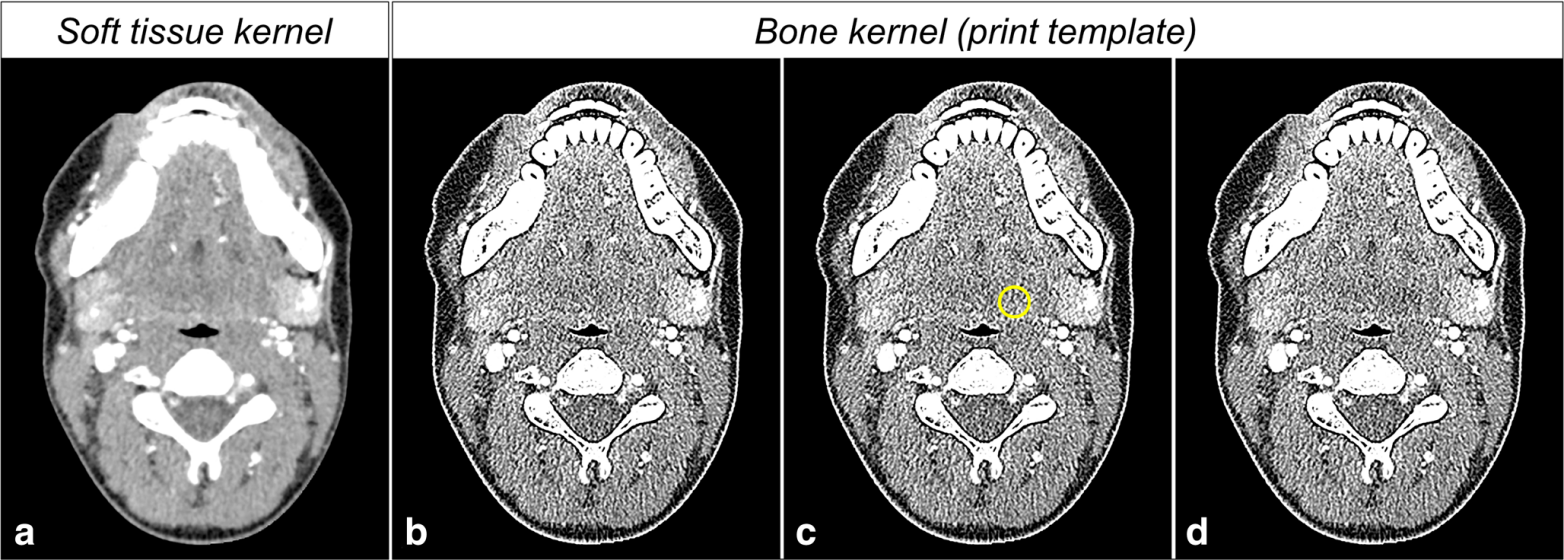
Manipulation of a CT image as a print template. **a** CT image of a patient (soft tissue kernel). **b** Bone kernel reconstruction of the same image. **c** Circular region of interest of 1 cm diameter in the left parapharyngeal space. **d** Manipulated image with a hypodense lesion of 40 HU contrast

A contrast medium‑enhanced CT image of a patient’s neck was retrospectively selected from our clinical database. The image was acquired with a Canon Acquilion Prime CT system (Canon Medical Systems) and reconstructed with 0.8-mm slice thickness, a bone kernel (FC30), and adaptive iterative dose reduction 3D (AIDR-3D). The reconstruction parameters were selected based on preliminary work in preparation for radiopaque inkjet printing (described further below). The image was manipulated to create four additional images with low-contrast lesions of 1 cm diameter and 10, 20, 30, and 40 Hounsfield unit (HU) contrast. To this end, open-source software (Horos Project) was used to subtract 10, 20, 30, and 40 HU from every pixel inside a circular region of interest (ROI) of 1 cm diameter in the left parapharyngeal space. The ROI size was selected to create lesions that most radiologists would consider to be clinically relevant and within the scope of their diagnostic work. The ROI position was not changed between images to provide identical conditions for the subsequent analysis of the lesions. Pixelwise subtraction was used to reduce HU without changing the texture inside the ROI. In summary, five DICOM images were prepared as templates for radiopaque inkjet printing: the original patient image and four manipulated versions of the same patient image with low-contrast lesions of 10, 20, 30, and 40 HU contrast in the same position in all images. Figure 1 shows a soft tissue kernel reconstruction of the patient image and illustrates how lesions were created in the print template (bone kernel reconstruction).

### Phantom construction

Radiopaque inkjet printing and paper-based 3D printing were used to create phantoms from the template image files [8, 9]. In preparation for the inkjet printing step, the template images were processed using a gray-scale correction procedure as previously described [8]. Each of the five resulting image files was repeatedly printed on 143 paper sheets (70 g/m^2^) using inkjet printing with potassium-iodide-doped ink (600 mg/mL) as previously described [8]. The paper was coated with a polyethylene film of 8 g/m^2^, which served as a thermoplastic adhesive, similar to the toner used in previous work [9]. The resulting five paper stacks, each consisting of 143 repeated prints of the same template image, were assembled to create phantoms of 1 cm thickness and cut to the patient’s neck shape using paper-based 3D printing [9]. Five phantoms were created in this way—four containing low-contrast lesions and one containing no lesion. The lesions had a rod shape, which resulted from stacking repeated prints of the same template images. The lesions had lower iodine content than the surrounding tissue due to reduced ink deposition by the inkjet printer as a result of HU subtraction from the template images.

### Analysis of lesion contrast

The phantoms were imaged with a Canon Aquilion Prime CT scanner. Twenty-seven acquisitions were performed with different CT settings (Table 1). The settings were varied to analyze lesion contrasts and their impact on detectability across multiple scan parameters. Images were reconstructed with 0.5 mm slice thickness and a soft tissue kernel (FC08). Two image data sets were created per acquisition using filtered back projection (FBP) and adaptive iterative dose reduction 3D (AIDR-3D) in a standard mode. Thirteen images per data set and phantom were extracted. ROIs of 0.5 cm^2^ and 3 cm^2^ were placed inside and around the low-contrast lesions, respectively. Mean HU values were analyzed and the HU difference between the ROIs was calculated to determine contrast values.

**Table 1:** CT acquisition settings. All possible combinations of two tube voltage settings, six tube current settings, and three pitch settings were used for image acquisition. Automatic tube potential selection (ATPS) recommended 100 kVp for all acquisitions and was only selectable in combination with automated tube current modulation (ATCM)

Tube voltage	Tube current	Pitch
▪ ATPS (100 kVp) ▪ 120 kVp	▪ ATCM SD 14 ▪ ATCM SD 10 ▪ ATCM SD 7.5 ▪ 150 mA ▪ 200 mA ▪ 250 mA	▪ 0.637 ▪ 0.813 ▪ 1.388

### Detectability experiment

**Fig. 2:**
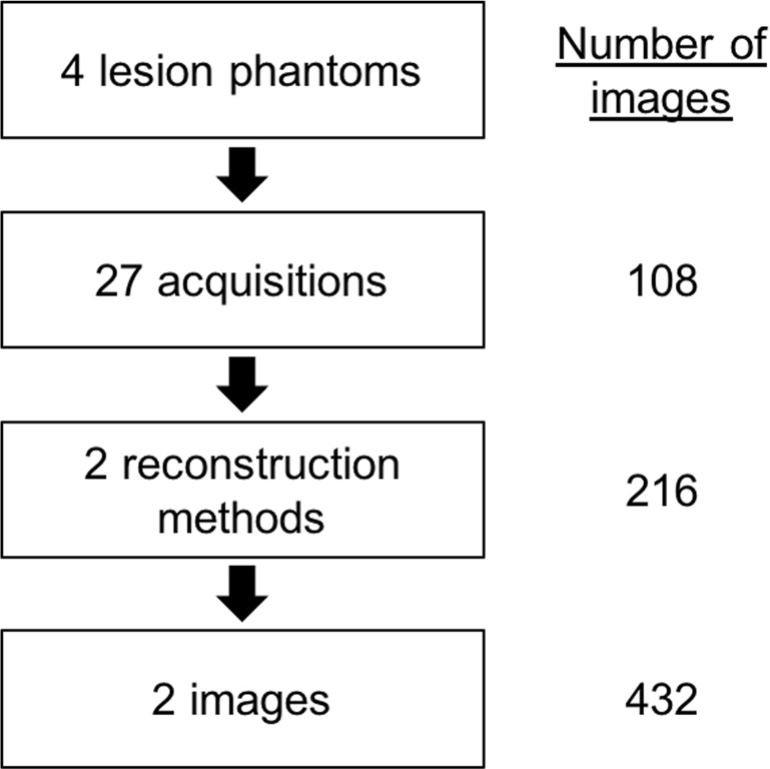
Flowchart illustrating how lesion images were generated and extracted for the detectability experiment. Every lesion image was paired with a non-lesion image that was acquired and reconstructed with the same CT settings

Two images were extracted from each of the 54 data sets of each of the five phantoms (with 10, 20, 30, and 40 HU lesion contrast and one without any lesion). Each image containing a lesion was paired with a non-lesion image that was acquired and reconstructed with the same CT settings. The image pairs were randomized and presented to seven blinded radiologists in a 2-alternative forced choice (2-AFC) experiment. Every reader was presented with a total of 432 image pairs (Fig. 2). A reference drawing indicating the position of the expected lesion was additionally displayed throughout the experiments. For every image pair, readers were asked to indicate which image contained a lesion. They were also asked to indicate their confidence using a five-step scale (1 = unconfident, 2 = rather unconfident, 3 = intermediate, 4 = rather confident, 5 = confident). Readers were instructed to assign a confidence level of 1 when they merely guessed and a confidence level of 5 when they were absolutely confident about their selection.

### Data analysis

Measured Hounsfield units are presented as mean ± standard deviation (SD) and as median and range. Detection accuracy was calculated as the percentage of correct lesion selections per reader. Results were compared using *t* tests and analysis of variance for repeated measurements with Tukey post hoc tests. Differences were interpreted as significant when *p* < 0.05.

## Results

### Phantoms

**Fig. 3:**
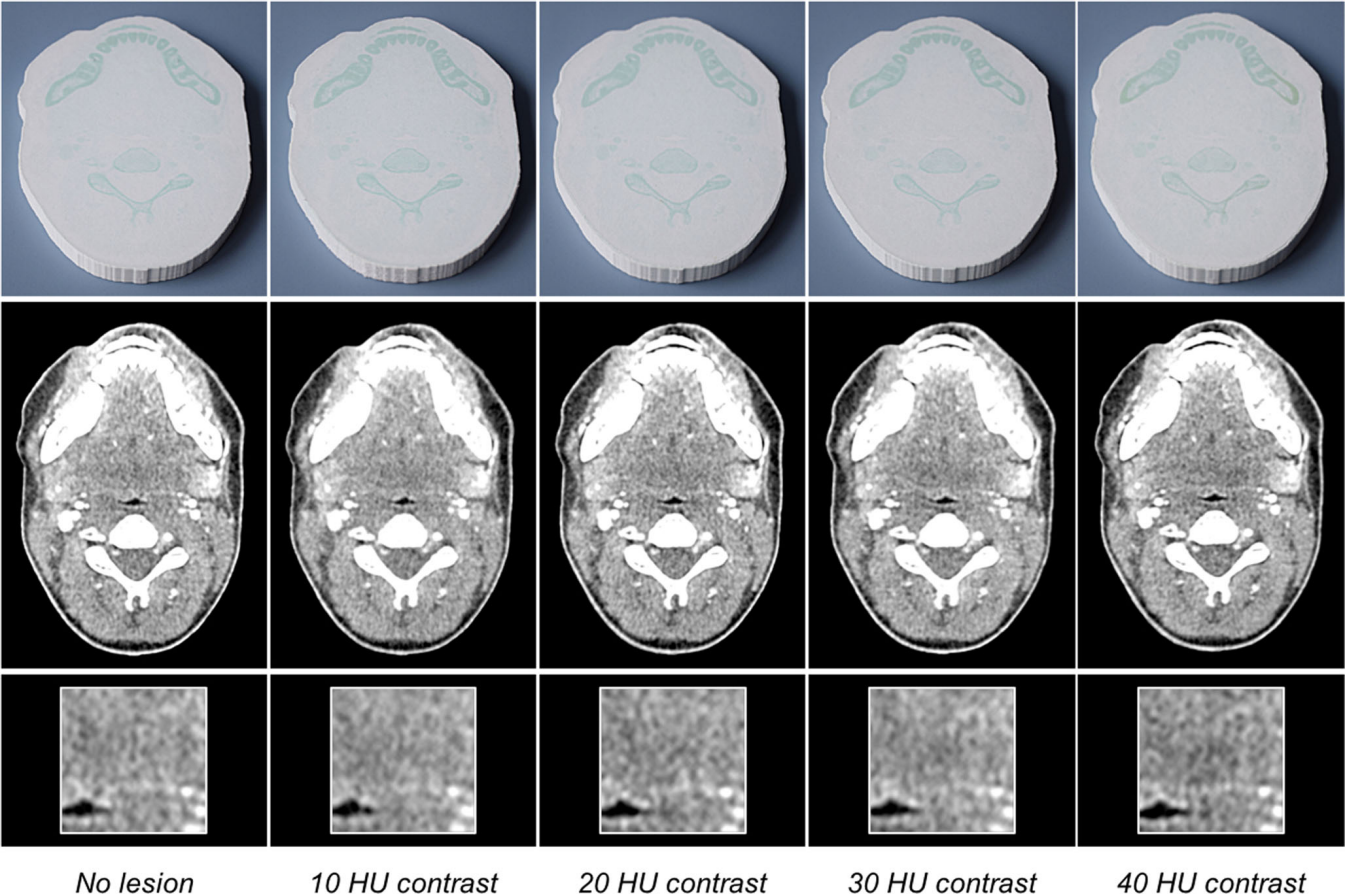
Photographs and CT images of the phantoms. The bottom row shows magnified details of the lesions in the left parapharyngeal space. All images are displayed with window level 40 and window width 350

Figure 3 shows photographs and CT images of the phantoms and the original patient. All images are displayed with window level 40 and window width 350. The phantoms reproduced the size and the shape of the patient (mean ± SD phantom area 130.9 ± 0.1 cm^2^ vs. 131 cm^2^ for the patient).

### Lesion contrast

**Fig. 4:**
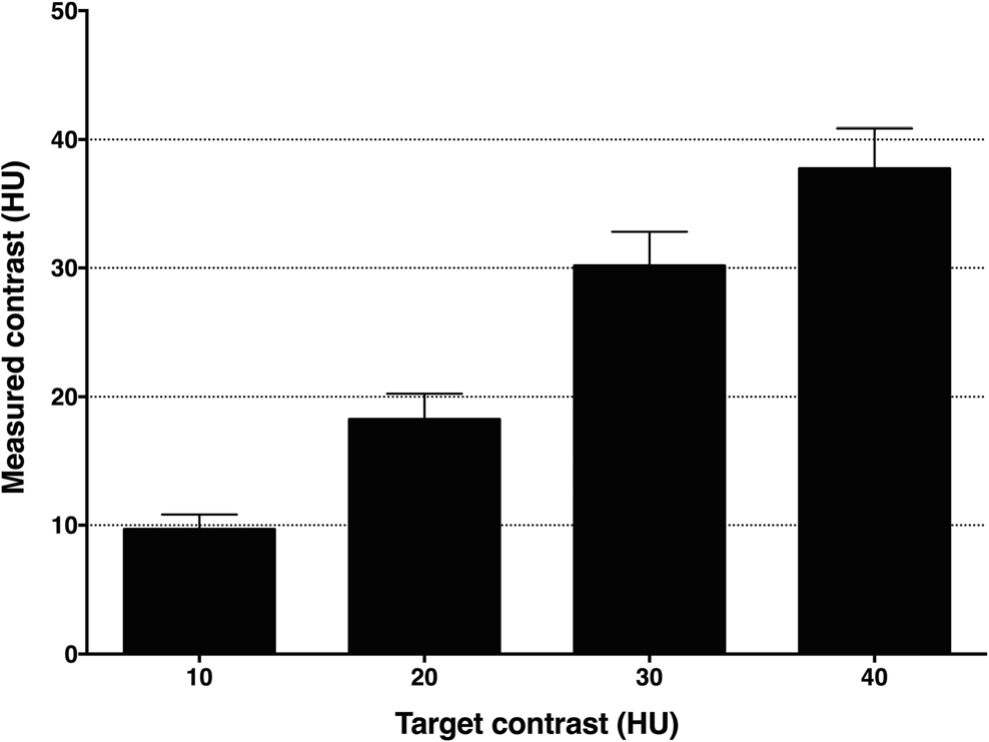
Target and measured lesion contrasts. Means and standard deviations for 54 CT acquisitions are shown

Measured mean ± SD contrast values of the low-contrast lesions were 9.7 ± 1.2 HU for the 10 HU contrast sample, 18.2 ± 2 HU for the 20 HU contrast sample, 30.2 ± 2.7 HU for the 30 HU contrast sample, and 37.7 ± 3.1 HU for the 40 HU contrast sample (Fig. 4). Detailed results for all acquisitions are provided in Suppl. Table 1. There was a significant increase in contrast for all samples with 100-kVp tube voltage compared with 120-kVp tube voltage (*p* < 0.023).

### Detectability experiment

**Fig. 5:**
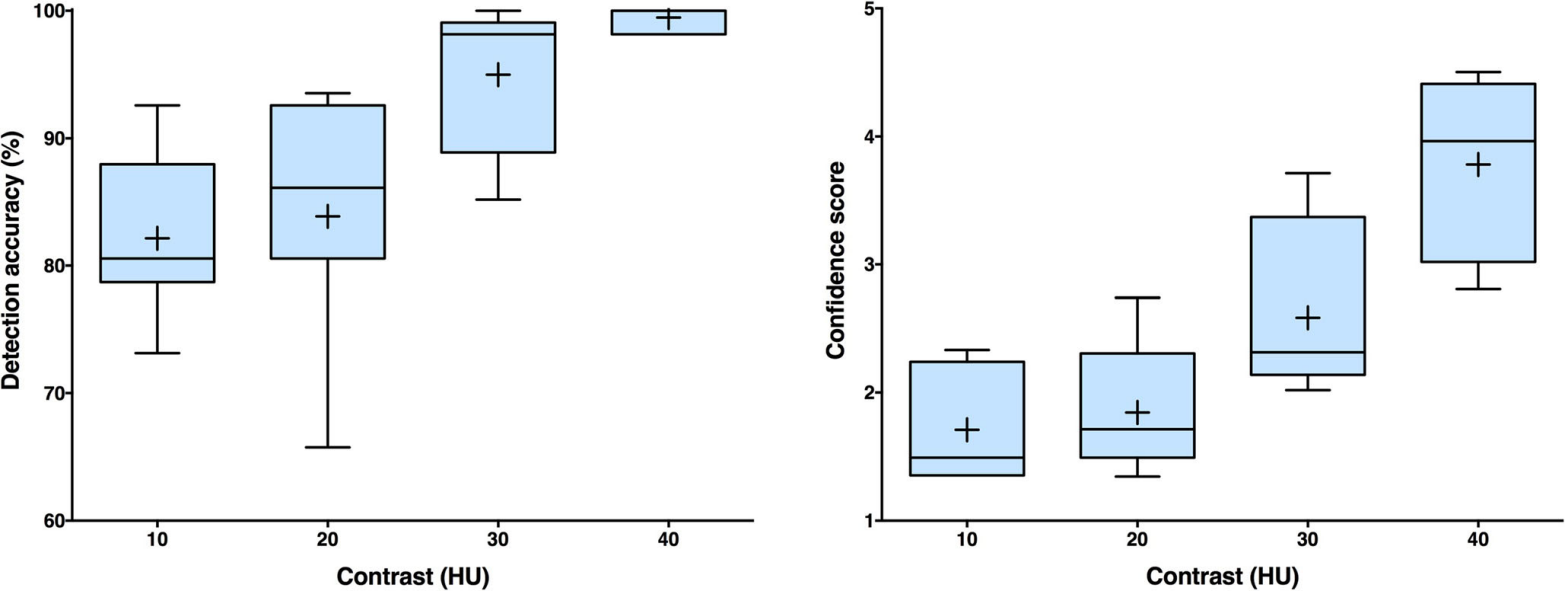
Detection accuracies (left) and confidence scores (right). Reader results for seven radiologists are shown

Figure 5 shows the detection accuracy and diagnostic confidence results from the detectability experiment. Mean ± SD detection accuracy values across the seven radiologists were 82.1 ± 6.3% for 10 HU lesion contrast, 83.9 ± 9.4% for 20 HU contrast, 95 ± 5.7% for 30 HU contrast, and 99.5 ± 0.9% for 40 HU contrast. The increase in detection accuracy was statistically significant between 20 and 30 HU contrast (*p* = 0.007) and not significant between 10 and 20 HU contrast (*p* = 0.863) and between 30 and 40 HU contrast (*p* = 0.231). The readers’ confidence scores were low and not significantly different for 10 and 20 HU lesion contrast (1.71 ± 0.41 and 1.84 ± 0.5, *p* = 0.159). They significantly increased with lesion contrast to 2.59 ± 0.67 for 30 HU contrast and 3.78 ± 0.66 for 40 HU contrast (*p* < 0.005).

## Discussion

Detectability experiments use detection tasks to assess the diagnostic performance of CT images and should mimic the clinical situation realistically. To this end, anatomically realistic phantoms with low-contrast lesions of 10 to 40 HU contrast were developed and lesion contrasts and their impact on detectability by radiologists were evaluated. The developed approach creates a groundwork for the assessment of CT performance with methods that mimic the clinical work of radiologists.

Good agreement between target and measured lesion contrast values was achieved because gray scales of the printed images were correlated linearly with printer ink deposition and resulting HU values as previously described [8]. Variations in contrasts measured with different scanner settings were expected, as CT settings were previously shown to affect CT numbers [10, 11]. The observed contrast increase at a lower tube voltage was reinforced by the iodine content of the phantoms. The results are in line with previous observations in phantoms and patients after contrast medium administration [12–14], underlining that the phantoms simulate the clinical situation adequately. Contrast increase at reduced tube voltage is less pronounced for soft tissues without contrast medium enhancement [15], which may thus limit the suitability of the phantoms for studying tube voltage effects in situations where CT scans are acquired without contrast medium administration.

Detection accuracy and confidence scores increased significantly from 20 to 30 HU lesion contrast, but not from 10 to 20 HU contrast as would have been expected from previous studies using similar contrast levels [16, 17]. However, these previous studies used uniform phantoms, and the results of the present study may thus be explained by the anatomical texture of the phantoms. This conclusion is to some extent supported by a previous model observer study, which reported detectability to increase between 10 and 14 HU lesion contrast for a uniform phantom, but not for a textured phantom with small-scale features and only slightly for two other textured phantoms with larger-scale features [7]. However, comparability of our findings with this study is also limited because different phantoms, lesion sizes, and scan settings were used and because observer variability is lower for model observers than for human observers as in the present study.

Remarkably, the detection accuracy results for 10 and 20 HU contrast lesions were relatively high despite the low contrast and reader confidence. This can be explained by the location-known-exactly experimental design, where the task was rather simple as the participating radiologists were aware of the expected lesion position [18]. Future work on evaluating CT techniques with the methodology presented here should consider a search task with lesions in unknown locations. The aim of the present study was to provide a groundwork for such studies by developing anatomical phantoms for detection tasks and providing an estimate of reasonable lesion contrast across different scanner settings to be used in such studies. The results suggest a contrast of 20 to 30 HU, where the participants’ confidence and detection success changed most significantly in the 2-AFC experiment.

The present study did not address the relationship between CT scan settings and resulting dose exposure and detectability scores. Studies aimed at investigating these relationships should consider that the scan length affects the dose-length product (DLP), notably the contribution of overscanning to DLPs with different pitch values. Furthermore, it should be considered that scan length and anatomical variation also affect tube current modulation behavior and resulting doses. For a realistic setup in such studies, the phantoms presented here could be inserted between anatomically realistic parts of a head-and-neck phantom. It could also be considered to provide data sets that study participants can scroll through. However, the appearance of the rod-shaped lesions would not change between images and the participants would be required to evaluate substantially more images, which is why scrolling was not considered for reading in the present study.

Previous work evaluated low-contrast lesions in cylindrical phantoms with textured background [7] or in CT images with digitally inserted lesions [19, 20]. However, to the authors’ knowledge, no previous work created anatomically realistic phantoms with low-contrast lesions. Such phantoms have the advantage of simulating the entire diagnostic process that patients undergo. They can repeatedly be scanned with the same or different CT systems to study inter- and intrascanner variations and acquisition techniques. The phantom images are similar to clinical images and can be used to perform detection tasks that are similar to clinical tasks of radiologists. They thus offer novel possibilities for investigating image quality more realistically than with uniform phantoms and for studying systematic scan parameter variation, which is precluded in clinical trials.

The limitations of this study include that only one patient was simulated and that only one lesion size was used. Detectability results may differ in other anatomical regions and with smaller or larger lesions. Conclusions regarding the influence of background texture are limited because there was no direct comparison with uniform phantoms. The impact of scan settings on dose and lesion detectability was beyond the scope of this work and therefore not analyzed. Also, the results we report here apply only to the CT system and the CT settings that were used in the present work.

The method we report here for the creation of phantoms to be used for detection tasks enables CT image quality to be evaluated with images and methods that mimic the clinical practice of radiologists. This is of relevance for a broad range of clinical and scientific applications including CT protocol optimization and the assessment of novel CT techniques. Such patient-mimicking phantoms have the potential to reduce patient exposure in clinical trials and to accelerate CT optimization for safer diagnostic patient imaging.

## Electronic supplementary material

ESM 1 (DOCX 26 kb)

## Abbreviations

2-AFC: 2-Alternative forced choice
AIDR-3D: Adaptive iterative dose reduction 3D
ATCM: Automated tube current modulation
ATPS: Automatic tube potential selection
CNR: Contrast-to-noise ratio
CT: Computed tomography
FBP: Filtered back projection
HU: Hounsfield unit
IR: Iterative reconstruction
ROI: Region of interest
SD: Standard deviation
