# Supplementary material for: 3D printing of anatomically realistic phantoms with detection tasks to assess the diagnostic performance of CT images
Source: Eur Radiol. 2020 Mar 28;30(8):4557–63. doi: 10.1007/s00330-020-06808-7 (PMC7338819; doi:10.1007/s00330-020-06808-7)
Supplement: Supplementary file 1 — (DOCX 26 kb) [file 330_2020_6808_MOESM1_ESM.docx]

Suppl. table 1: Measured lesion contrast values of fifty-four CT acquisitions. Mean ± standard deviation values are provided.

| **Tube voltage** | **Tube current** | **Pitch** | **Reconstruction** | **10 HU lesion contrast** | **20 HU lesion contrast** | **30 HU lesion contrast** | **40 HU lesion contrast** |
| --- | --- | --- | --- | --- | --- | --- | --- |
| ATPS (100 kVp) | ATCM SD 7.5 | 0.813 | FBP | 11.3±2.6 | 19.1±2.2 | 34.5±4.1 | 41.1±2.0 |
| ATPS (100 kVp) | ATCM SD 10 | 0.813 | FBP | 10.8±4.6 | 18.9±4.7 | 35.2±3.5 | 41.8±3.2 |
| ATPS (100 kVp) | ATCM SD 14 | 0.813 | FBP | 9.7±4.7 | 20.9±4.3 | 32.7±5.6 | 42.3±4.7 |
| ATPS (100 kVp) | ATCM SD 7.5 | 0.637 | FBP | 9.5±2.5 | 19.8±2.1 | 32.9±2.9 | 40.6±2.2 |
| ATPS (100 kVp) | ATCM SD 10 | 0.637 | FBP | 10.6±4.8 | 19.8±3.7 | 32.3±2.7 | 42.4±3.6 |
| ATPS (100 kVp) | ATCM SD 14 | 0.637 | FBP | 10.0±5.2 | 17.5±4.6 | 33.4±3.6 | 41.9±4.6 |
| ATPS (100 kVp) | ATCM SD 7.5 | 1.388 | FBP | 9.1±2.3 | 22.4±1.8 | 32.5±3.9 | 43.9±4.1 |
| ATPS (100 kVp) | ATCM SD 10 | 1.388 | FBP | 7.8±2.3 | 23.7±6.2 | 32.1±2.1 | 40.8±2.2 |
| ATPS (100 kVp) | ATCM SD 14 | 1.388 | FBP | 9.1±4.3 | 21.3±2.4 | 34.8±5.1 | 45.1±6.2 |
| 120 kVp | ATCM SD 7.5 | 0.813 | FBP | 10.0±2.2 | 18.3±1.2 | 28.1±2.0 | 36.4±2.1 |
| 120 kVp | ATCM SD 10 | 0.813 | FBP | 7.9±4.5 | 17.6±2.1 | 27.9±2.8 | 36.0±2.7 |
| 120 kVp | ATCM SD 14 | 0.813 | FBP | 8.2±4.0 | 18.0±4.7 | 28.5±3.1 | 37.7±3.2 |
| 120 kVp | ATCM SD 7.5 | 0.637 | FBP | 10.3±2.2 | 19.0±1.9 | 28.5±1.4 | 34.9±2.7 |
| 120 kVp | ATCM SD 10 | 0.637 | FBP | 9.5±2.3 | 17.9±2.3 | 27.6±2.8 | 34.0±2.8 |
| 120 kVp | ATCM SD 14 | 0.637 | FBP | 9.1±3.2 | 18.1±2.4 | 28.7±2.2 | 36.2±2.8 |
| 120 kVp | ATCM SD 7.5 | 1.388 | FBP | 11.6±3.6 | 17.8±3.0 | 28.1±2.4 | 35.2±1.5 |
| 120 kVp | ATCM SD 10 | 1.388 | FBP | 9.1±1.8 | 18.3±1.9 | 25.5±1.8 | 35.0±1.9 |
| 120 kVp | ATCM SD 14 | 1.388 | FBP | 9.5±2.2 | 16.8±2.2 | 27.4±3.4 | 38.2±4.0 |
| 120 kVp | 150 mA | 0.813 | FBP | 10.0±2.2 | 17.0±1.7 | 29.6±1.7 | 36.2±2.1 |
| 120 kVp | 200 mA | 0.813 | FBP | 9.2±2.9 | 16.1±1.1 | 28.9±1.8 | 35.3±2.2 |
| 120 kVp | 250 mA | 0.813 | FBP | 10.3±2.5 | 17.8±2.1 | 27.7±2.7 | 35.6±2.6 |
| 120 kVp | 150 mA | 0.637 | FBP | 9.5±2.2 | 17.0±1.6 | 29.9±3.0 | 36.7±3.0 |
| 120 kVp | 200 mA | 0.637 | FBP | 8.2±1.5 | 18.0±1.8 | 30.1±2.0 | 36.3±2.1 |
| 120 kVp | 250 mA | 0.637 | FBP | 8.3±2.8 | 17.6±1.2 | 28.5±2.5 | 36.3±2.5 |
| 120 kVp | 150 mA | 1.388 | FBP | 7.6±2.0 | 17.9±2.2 | 26.1±2.7 | 35.7±1.7 |
| 120 kVp | 200 mA | 1.388 | FBP | 10.7±2.0 | 14.7±3.4 | 29.6±1.7 | 37.7±2.2 |
| 120 kVp | 250 mA | 1.388 | FBP | 7.3±4.4 | 15.1±1.6 | 29.8±2.9 | 33.2±1.4 |
| ATPS (100 kVp) | ATCM SD 7.5 | 0.813 | AIDR-3D | 11.6±1.9 | 18.3±1.7 | 34.9±2.5 | 41.0±1.7 |
| ATPS (100 kVp) | ATCM SD 10 | 0.813 | AIDR-3D | 11.1±2.1 | 18.7±1.5 | 35.3±2.1 | 40.4±3.1 |
| ATPS (100 kVp) | ATCM SD 14 | 0.813 | AIDR-3D | 10.8±2.9 | 21.0±2.3 | 34.0±1.8 | 41.7±3.1 |
| ATPS (100 kVp) | ATCM SD 7.5 | 0.637 | AIDR-3D | 10.7±2.4 | 19.3±1.1 | 32.7±1.7 | 40.2±1.7 |
| ATPS (100 kVp) | ATCM SD 10 | 0.637 | AIDR-3D | 10.4±3.1 | 19.7±1.3 | 32.2±0.9 | 40.6±1.5 |
| ATPS (100 kVp) | ATCM SD 14 | 0.637 | AIDR-3D | 11.7±2.4 | 15.5±1.6 | 32.7±1.5 | 39.6±1.8 |
| ATPS (100 kVp) | ATCM SD 7.5 | 1.388 | AIDR-3D | 10.2±2.4 | 22.9±1.6 | 33.0±3.3 | 43.4±3.4 |
| ATPS (100 kVp) | ATCM SD 10 | 1.388 | AIDR-3D | 8.7±2.5 | 22.3±5.9 | 32.6±1.8 | 40.3±1.9 |
| ATPS (100 kVp) | ATCM SD 14 | 1.388 | AIDR-3D | 10.2±2.9 | 21.8±1.4 | 35.0±4.8 | 43.7±5.0 |
| 120 kVp | ATCM SD 7.5 | 0.813 | AIDR-3D | 10.6±2.5 | 17.7±1.4 | 28.2±1.6 | 36.2±1.8 |
| 120 kVp | ATCM SD 10 | 0.813 | AIDR-3D | 8.0±3.4 | 17.6±1.2 | 28.4±2.1 | 35.5±2.0 |
| 120 kVp | ATCM SD 14 | 0.813 | AIDR-3D | 9.4±2.6 | 17.9±1.5 | 28.6±1.5 | 37.1±2.4 |
| 120 kVp | ATCM SD 7.5 | 0.637 | AIDR-3D | 11.2±1.7 | 18.7±1.4 | 29.1±1.2 | 35.1±2.1 |
| 120 kVp | ATCM SD 10 | 0.637 | AIDR-3D | 10.2±2.5 | 17.4±1.3 | 27.8±1.2 | 33.6±1.6 |
| 120 kVp | ATCM SD 14 | 0.637 | AIDR-3D | 9.4±2.5 | 16.8±1.3 | 28.0±0.9 | 34.8±2.6 |
| 120 kVp | ATCM SD 7.5 | 1.388 | AIDR-3D | 11.4±3.8 | 16.9±2.7 | 28.5±2.1 | 35.6±1.4 |
| 120 kVp | ATCM SD 10 | 1.388 | AIDR-3D | 9.7±2.1 | 17.9±1.5 | 25.6±1.6 | 34.3±1.3 |
| 120 kVp | ATCM SD 14 | 1.388 | AIDR-3D | 9.4±2.1 | 16.9±1.7 | 28.1±1.7 | 37.2±3.1 |
| 120 kVp | 150 mA | 0.813 | AIDR-3D | 10.2±1.8 | 17.1±1.2 | 30.1±1.1 | 35.9±1.8 |
| 120 kVp | 200 mA | 0.813 | AIDR-3D | 9.2±2.6 | 15.8±1.1 | 29.4±1.4 | 35.3±1.7 |
| 120 kVp | 250 mA | 0.813 | AIDR-3D | 10.4±2.4 | 17.7±1.8 | 28.3±2.2 | 35.3±2.6 |
| 120 kVp | 150 mA | 0.637 | AIDR-3D | 10.2±2.2 | 17.0±0.8 | 30.0±2.5 | 36.5±2.2 |
| 120 kVp | 200 mA | 0.637 | AIDR-3D | 8.5±1.4 | 17.9±1.7 | 30.2±1.7 | 36.2±1.6 |
| 120 kVp | 250 mA | 0.637 | AIDR-3D | 8.4±2.4 | 17.6±1.2 | 28.8±2.2 | 36.0±2.1 |
| 120 kVp | 150 mA | 1.388 | AIDR-3D | 8.3±2.3 | 17.7±1.3 | 26.3±2.2 | 35.5±1.5 |
| 120 kVp | 200 mA | 1.388 | AIDR-3D | 10.9±1.6 | 15.0±2.9 | 29.6±1.0 | 37.2±1.8 |
| 120 kVp | 250 mA | 1.388 | AIDR-3D | 7.3±4.5 | 15.3±1.1 | 30.5±1.9 | 33.0±0.9 |
